# Improvements to the living environment and mental health among older adults: an empirical study based on the Chinese experience

**DOI:** 10.3389/fpubh.2026.1850493

**Published:** 2026-07-28

**Authors:** Guangjin Zhong, Mingyu Zhou, Shiyao Peng

**Affiliations:** 1 College of Public Administration and Law, Hunan Agricultural University, Changsha, Hunan, China; 2 Changsha Ruisheng Testing Technology Co., Ltd., Changsha, Hunan, China

**Keywords:** improvements to the living environment, mental health of older adults, sleep quality, social participation, future confidence

## Abstract

**Background:**

Against the backdrop of global aging and sustainable development, China is confronted with the dual challenges of improving mental health among the older adults and enhancing their living environment.

**Methods:**

Based on data from the 2020 China Longitudinal Study of Aging, this study employs an ordinary least squares regression model to investigate the impact of improvements to the living environment on the mental health of the older adults and its underlying mechanisms.

**Results:**

The results indicate that improvements to the living environment can extend beyond the realm of physical health and exert significant positive effect on the mental health of older adults. Every one-unit increase in environmental satisfaction reduces the older adults’ depression scores by 0.426 points. Mechanism analysis indicates that improvements to the living environment enhance the mental health of older adults through three parallel pathways: improving sleep quality (physiological pathway), promoting social participation (behavioral pathway), and strengthening confidence in the future (cognitive pathway). However, the psychological benefits of improvements in the living environment are constrained by three factors—urban–rural disparities, economic income, and personal health, these benefits are more pronounced among three specific groups of older adults: those in rural areas, those with high-income, and those who self-reported good health.

**Conclusion:**

Consequently,future environmental policies should prioritize the synergistic benefits of mental health, and incorporate them into performance evaluation frameworks. This will better facilitate the channeling of policy resources toward groups with limited access to such benefits, further foster an age-friendly social environment, and ultimately promote the deep integration of improvements to the living environment with active aging.

## Introduction

In the global pursuit of sustainable development, intensifying climate extreme, deteriorating air quality, and marine pollution pose severe challenges to the Earth’s ecosystems. The living environment, which forms the foundation for consolidating public health and people’s well-being, has gradually become a focal point of international attention. In September 2015, the United Nations Sustainable Development Summit formally adopted the 17 Sustainable Development Goals (SDGs). It explicitly establishes close linkages between Good Health and Well-being and environmental dimensions including “Clean Water and Sanitation,” “Sustainable Cities and Communities,” and “Climate Change,” thereby highlighting the vital role of a sound ecological environment as a fundamental prerequisite for human health. Against this backdrop, the “One Health” concept, which emphasizes the close connection and interdependence of human health, animal health, and ecosystem health, has established a theoretical framework for revealing the health benefits of improving the living environment. Only by mobilizing multiple sectors, disciplines and communities at various levels of society and strengthening collaborative governance of diverse stakeholders can we enhance social well-being and address threats to health and ecosystems. As an active participant, significant contributor, and pacesetter in global ecological civilization construction, China firmly upholds the concept of a community with a shared future for mankind. While pursuing the process of a Beautiful China, China adheres to multilateralism and consistently contributes tangible Chinese solutions to global environmental governance through concrete actions. It has continuously waged a campaign to combat pollution, centered on “Protecting Blue Skies, Clear Waters and Clean Land.” After years of rigorous governance, the quality of ambient air, cultivated land and water environments has been comprehensively improved, laying a solid foundation for perfecting the ecological and environmental governance system and fuether implementing the Healthy China Initiative. Nevertheless, China is confronting multiple social and health challenges stemming from rapid population aging. The size of older adults population continues to expand, accompanied by an accelerating trend of advanced aging and empty-nest households. Psychological issues such as depression and anxiety among older adults are increasingly prevalent, constituting a major hidden hazard impairing their quality of life and health well-being. By the end of 2024, China’s population aged 60 and above had risen to 310 million, accounting for 22% of the total population, indicating that China has officially entered a moderately aged society. Among this group, 23.76 and 26.40% of older adults experience varying degrees of loneliness and depression, respectively. Addressing mental health issues among older adults has thus become an essential approach to enhancing their well-being in later life. Existing studies have found that environmental exposures such as air pollution, noise disturbance, and climate change exacerbate psychological distress among older adults via physiological stress, behavioral restrictions and reduced social interaction, whereas environmental governance exerts positive effects on their mental health by improving the quality of the living environment and promoting social engagement and interpersonal communication. On this basis, this paper aims to move beyond the traditional perspective of physical health to deeply explore the impact effects and mechanisms through which the living environment influences the mental health of older adults. It seeks to expand the application of the “One Health” concept in the interdisciplinary field of environmental science and public health, provides a theoretical basis for realizing the health dividends of environmental policies in an aging population, and promote the deep integration of the “Healthy China 2030” initiative and the global Sustainable Development Goals.

## Literature review

In the 1970s, the European Community began formulated and implemented the “Environmental Action Programmes” designed to improve environmental quality (1), and gradually assumed a leading role in global environmental governance agenda. Since the founding of the People’s Republic of China, the Communist Party of China and the Central People’s Government have attached great importance to safeguarding the production and living environments of the general public; however, formal environmental governance emerged almost simultaneously with the rise of the worldwide environmental movements. Specifically, the evolution of China’s environmental governance can be divided into three phases: the planned economy period, the socialist market economy period, and the new era of socialism with Chinese characteristics. In the initial stage of environmental governance, environmental protection was enshrined as a fundamental national policy of China, adhering to the guideline that environmental development, economic development and urban–rural development shall be planned, implemented and advanced in tandem (2). In October 1992, the 14th National Congress of the Communist Party of China formally set out to establish a socialist market economic system, and environmental protection was elevated to a development objective of equal importance to economic growth. The 17th National Congress of the Communist Party of China further identified ecological civilization construction as a new requirement for achieving the goal of building a moderately prosperous society in all respects. Since the entry into the new era of socialism with Chinese characteristics, the modernization of environmental governance has become an essential component and indispensable guarantee for the Chinese path to modernization.

In the early 21st century, the veterinarian William Karesh first coined the term “One Health” (3). The contemporary value of “One Health” lies in disseminating the philosophy of holistic health for all living things, which emphasizes the integrated health of humans, animals and the ecological environment. In recent years, two major Chinese strategies are consistent with the core values of “One Health.” The first is the sustainable development strategy. Sustainable development refers to the coordinated progress of the economy, society, resources and environmental protection. While pursuing economic benefits, it also emphasizes the protection of the natural resources and ecological environment upon which human survival depends, stressing harmonious coexistence between humanity and nature. This is precisely in line with the “One Health” concept of holistic health for humans, animals and nature. The second strategy is ecological civilization construction. As a fuether advance of the sustainable development strategy, the ecological civilization construction highlights that humans and nature form a community of life and dheres to the harmonious coexistence of humanity and nature (4). Environmental governance guided by the sustainable development trategies and ecological civilization construction generates significant health benefits. Environmental regulation improves the built environment of communities by reducing concentrations of pollutants such as sulfur dioxide, nitrogen oxides, and somke (or particulate) dust. It can not only effectively control the incidence and mortality rates of diseases highly correlated with air pollution levels but also alleviate the severity of diseases less sensitive to air pollution indirectly (5, 6). On the other hand, clean air initiatives help reduce both average length of hospital stays and hospitalization costs, thereby cutting overall healthcare expenditures. This effect is particularly pronounced in low- and middle-income cities and those with uneven medical insurance coverage (7). From the perspective of environmental psychology, there exists a reciprocal interplay between human activities, experiences and the environment. A high-quality community environment exerts a significant beneficial effect on older adults’ mental health (8), whereas adverse residential conditions including noise, dampness, inadequate lighting and poor hygiene can trigger a range of mental health issues (9), thereby increasing the risk of schizophrenia and suicide. Low- and middle-income countries are plagued by severe air pollution and climate change, and suffer from insufficient capacity to address mental health illnesses due to the scarcity of mental health service resources. Conversely, a favourable community environment can improve residents’ mental health by reducing the risk of depression and anxiety, accumulating social capital, and facilitating social participation (10, 11). Such environments generate more substantial gains in health capital for females, older adults and respondents with poor self-reported health, and also contribute to mitigating interregional health inequalities (12).

Regarding the measurement of mental health levels, Jorm AF et al. developed a scenario-based questionnaire based on the theory of mental health literacy (13); however, as this approach is limited by the specific scenarios, it needs to be used in conjunction with mental health scales. In 1905, Binet and Simon formally proposed the first psychometric scale, namely the Binet-Simon Intelligence Scale. In 1916, professor Lewis M. Terman, from Stanford University revised the Binet Scale and formulated detailed specifications on its use and scoring criteria (14). After the 1920s, the Woodworth Personal Data Form was developed. After World War II, numerous symptom-specific scales emerged, including the Beck Depression Inventory, the State–Trait Anxiety Inventory, and the Symptom Checklist 90. Although the measurement focuses of these scales vary, they generally assess an individual’s mental health across three dimensions: emotion, cognition and physiology. The development of Chinese psychological scales has undergone a transition from imported revision to independent innovation, gradually forming a tripartite structure: first, the Chinese Personality Assessment Inventory and the Chinese Values Scale, which are deeply rooted in traditional Chinese cultural philosophies; second, the Chinese College Students’ Mental Health Scale and the Center for Epidemiologic Studies Depression Scale (CES-D), which are closely linked to China’s social realities and prevalent physiology problem; and third, the Comprehensive Happiness Questionnaire and the Chinese Happiness Scale, which, based on a positive psychology perspective, emphasize the impact of harmonious interpersonal relationships on individual well-being.

In summary, existing researches on the relationship between improvements to the living environment and residents’ health has mainly concentrated on the physiological health dimension or examined the health effects of environmental regulations from a macro-policy perspective. Even though some studies have begun to address the impacts of the natural environment on mental health, the several limitations remain. First, empirical evidence linking residential environmental improvements to individual psychological changes requires further exploration. Second, few studies systematically analyze the mechanisms through which overall performance of the living environment improvements affects mental health; with regard to the vulnerable population of older adults, it is necessary to investigate the potential beneficial effects of environmental policies on their mental health through improvements in the ecological environment. Third, there is a lack of research on the heterogeneity of governance benefits across urban and rural areas, as well as among groups with vastly different income and health conditions. Based on this, this paper focuses on older adults population in China and transcends the conventional evaluation paradigm confined to physical health. It constructs an integrated analytical framework that incorporates living environment improvement and mental health effects. Adopting an “environment-psychology” interactive perspective to verify the mitigating effects of improvements to the living environment on psychological issues such as depression and anxiety, and to reveal the positive roles played by sleep quality, social connections and future optimism in this process. Concurrently, by incorporating demographic differences into the scope of this study, the paper analyses the heterogeneous effects of improvements to the living environment on mental health across different residential areas, income levels and self-reported health conditions. The aim is to accelerate the process of healthy aging through improvements to the living environment and to enrich research in the fields of ecological civilization and mental health.

## Theoretical framework and research hypotheses

**Figure 1:**
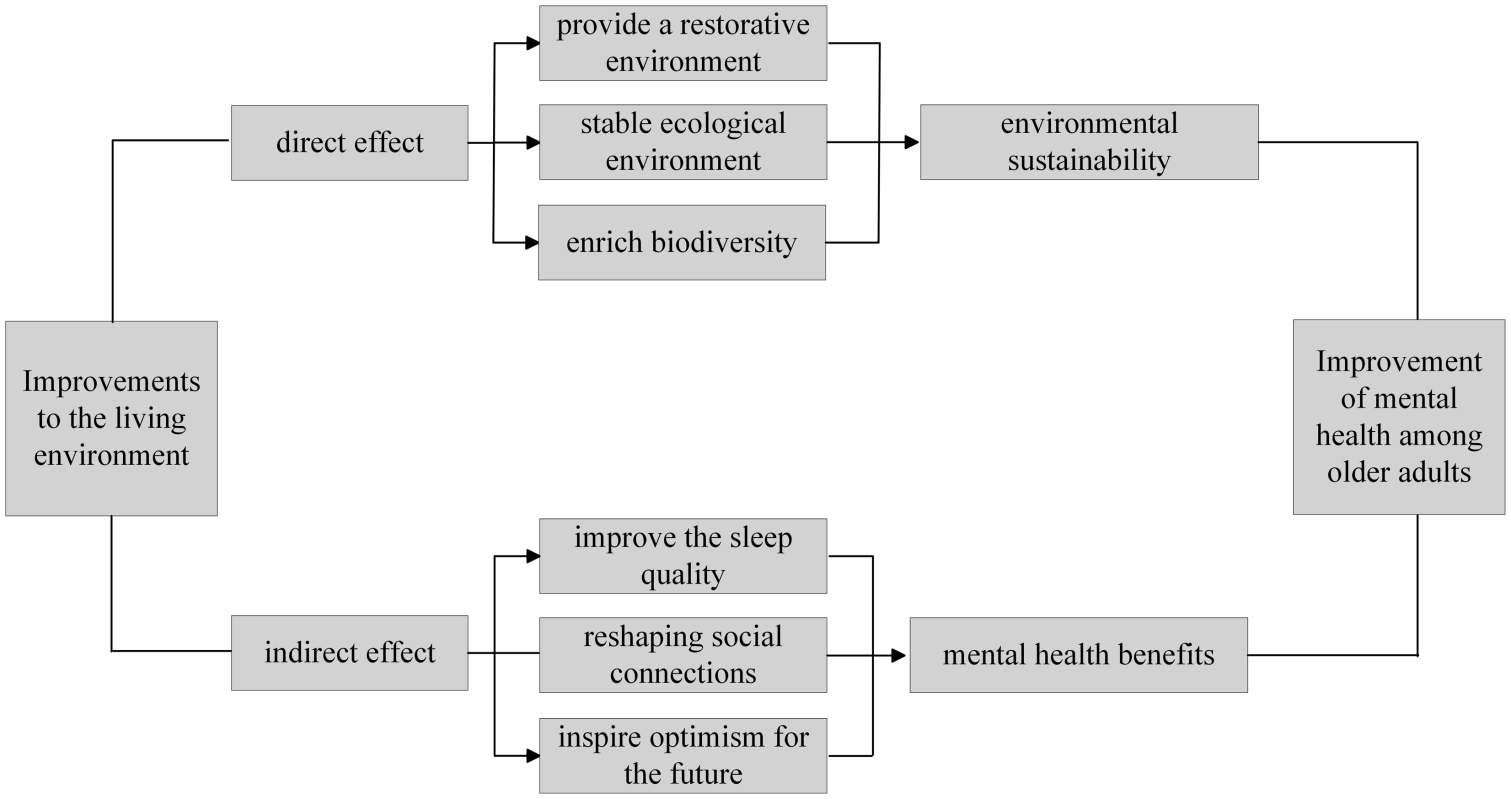
Analytical framework.

The “One Health” concept is a global strategic framework jointly advocated and promoted by the World Health Organization (WHO), the Food and Agriculture Organization of the United Nations (FAO), and the World Organisation for Animal Health (OIE). Its ideological origin can be traced back to the primitive understanding of the relationship between humans and nature in ancient medicine. The “One Health” concept in its modern sense has rapidly evolved in response to zoonoses (e.g., avian influenza and Ebola virus disease) and emerging infectious diseases, aiming to completely break down the long-standing disciplinary barriers between human medicine, veterinary medicine and environmental science. This concept views human health, animal health and environmental health as an interdependent and indivisible whole; the health status of any one component cannot exist independently of the other two, and an imbalance in any single link may trigger a health crisis across the entire system. The analytical framework is illustrated in Figure 1.

Environmental health, as a fundamental prerequisite for safeguarding human health, encompasses physical environmental safety, ecosystem stability and biodiversity conservation. First, improvements to the living environment contributes to the creation of a “restorative environment.” The products and services provided by a “restorative environment” represent an underutilize latent service function; after the older adults are exposed to, experience and interact with such environment, cognitive fatigue can be effectively alleviated. This ultimately translates into tangible psychological health benefits and improves individual health outcomes (15). Second, a stable ecological environment—such as stable climate and reduced natural disasters—positively influences individual mental health by reducing uncertainty risks, enhancing psychological security and improving life satisfaction (16). Third, biodiversity affects mental health by immune regulation and microbial exposure. Exposure to a diverse microbial environment helps enhance an individual’s immune regulatory functions, reduce chronic inflammation in the body, promote the synthesis, release, and metabolism of neurotransmitters, and decrease the secretion of stress hormones such as cortisol, thereby lowering the incidence risk of mental illnesses such as depression and anxiety. Accordingly, this paper proposes the following research hypotheses:

*H1:* Improvements to the living environment can transcend physiological boundaries to enhance the mental health of older adults.

The beneficial effects of improvements to the living environment on mental health of older adults are not transmitted through a single pathway, but rather exert indirect effects via multiple mediating mechanisms. Therefore, drawing upon the social support theory, environmental stress theory, and the broaden-and-build theory, this paper conducts a theoretical analysis of how improvements to the living environment influences older adults’ mental health from the micro-physiological, meso-behavioral, and macro-expectation perspectives.

Environmental stress theory emphasizes that negative stimuli in the physical environment impact an individual’s psychological system, thereby affecting their mental health, cognitive function and behavioral patterns (17). Sleep serves as a critical process for psychological recovery stressors such as noise and air pollution disrupt sleep architecture, leading to fragmented and shallow sleep, while sleep disorders are highly correlated with the risk of depression. Improvements to the living environment can effectively reduce negative stressors, thereby improving sleep efficiency, maintaining regular melatonin secretion, stabilizing the sleep–wake cycle, and strengthening the physiological foundation of mental health among older adults. Social support theory indicates that strong social networks can effectively mitigate the negative impact of stressful events on mental health (18). Networks characterized by high closeness, low heterogeneity, and numerous strong ties exert a positive influence on mental health. Public environments constitute an important spatial carrier for social interaction The optimization of public spaces including urban parks and community centers provides older adults with more opportunities for social engagement, thereby continuously expanding their social networks, accumulating social capital stock, and consequently strengthening their psychological resilience. The Broaden-and-Build Theory posits that positive emotions not only enhance individual well-being but also continuously broaden individual’s thought-action repertoires, thereby helping individuals maintain enduring social resources and yielding long-term adaptive benefits (19). By creating stable, safe, comfortable and accessible living surroundings, residential environment improvement provides objective settings for older adults to experience positive emotions. The accumulation of positive emotions reduces the risks of “isolation and loneliness” and “non-isolation but loneliness” among older adults, enabling them to maintain positive expectations for the future. Such accumulation encourages older adults to confront risks and challenges with a more flexible, open, and resilient attitude, thereby improving their mental health. Based on this, this paper proposes the following research hypothesis:

*H2:* A favorable living environment improves the mental health of older adults by enhancing sleep quality, rebuilding social connections, and stimulating future optimism.

## Research design

### Data sources

This study takes Chinese citizens aged 60 years and above as the research subjects. All data are derived from the 2020 China Longitudinal Study of Aging (CLASS). Designed and implemented by the Institute of Gerontology at Renmin University of China, this survey primarily employed a stratified multi-stage probability sampling method. The baseline survey covered 28 provinces and municipalities in China (excluding Hainan, Xizang, Xinjiang, Hong Kong, Macao, and Taiwan). The dataset provides solid empirical support for examining the relationship between living environment improvement and mental health among older Chinese adults. After eliminating outliers and merging certain variables, a total of 11,398 valid empirical samples were obtained.

### Model specification

Statistical analysis was conducted using Stata 17.0. Given that the mental health level of older adults is a continuous variable, this study employed an ordinary least squares (OLS) regression model to assess the impact of improvements in the living environment on the mental health of older adults, while also conducting robustness tests on the baseline regression results. Additionally, stepwise regression and Bootstrap sampling were used to examine, in particular, the mediating transmission mechanisms of sleep quality, social connections and future expectations. The basic model is shown below:

$$\text{menta}l_{i}=\beta_{1}\text{egoveranc}e_{i}+\beta_{2}x_{i}+\varepsilon_{i}$$

$$m_{i}=\beta_{3}\text{egoveranc}e_{i}+\beta_{4}x_{i}+\varepsilon_{i}$$

$$\text{menta}l_{i}=\beta_{5}\text{egoveranc}e_{i}+\beta_{6}m_{i}+\beta_{7}x_{i}+\varepsilon_{i}$$

In this model, *mental_i_* represents the dependent variable (mental health in older adults), *egovernance_i_* represents the independent variable (improvements to the living environment), *m_i_* represents the mediating variables (sleep quality, social connections and future expectations), and *x_i_* represents a set of control variables*_.(i)_* represents the random disturbance term. *β_1_*, *β_3_*, and *β_5_* are the estimated coefficients of the core explanatory variables, respectively; *β_6_* is the estimated coefficient of the mediating variable; *β_2_*, *β_4_*, and *β_7_* are the estimated coefficients of the control variables, respectively.

### Variable selection

Dependent variable: mental health in older adults. The Center for Epidemiological Studies Depression Scale (CES-D) was adopted to measure the mental health levels of the older adult population. The data were derived from respondents’ answers to the following questions in the CLASS questionnaire: “Do you feel in a good mood?,” “Do you feel lonely?,” “Do you feel sad?,” “Do you feel that life is going well?,” “Do you lose your appetite?,” “Do you feel useless?,” “Do you feel like you have nothing to do?,” and “Do you find much enjoyment in your life?.” The responses of “Never,” “Sometimes,” and “Often” are assigned scores of 0, 1 and 2, respectively. Among these items, “Do you feel in a good mood?,” “Do you feel that life is going well?,” and “Do you find much enjoyment in your life?” are reverse-scored, assigned 0, −1, and −2 points, respectively. The composite score ranges from [−6, 10], with higher scores indicating poorer psychological well-being among older adults. Life satisfation is used for robustness test in the subsequent analysis; the higher life satisfaction corresponds to better mental health status.

Independent variable: improvements to the living environment. Community environmental sanitation satisfaction can accurately and effectively reflect the actual impact of improvements to the living environment on the daily lives of older adults. Therefore, this study measures the level of improvement to the living environment based on older adults’ subjective perceptions and self-evaluations. The evaluating indicatort is constructed from respondents’ answers to the “Village (Community) Residents’ Satisfaction with Environmental Hygiene” section in the CLASS individual questionnaire, with “very satisfied,” “fairly satisfied,” “neutral,” “fairly dissatisfied,” and “very dissatisfied” assigned scores of 5, 4, 3, 2, and 1, respectively.

Mediating variables: sleep quality, social participation and future confidence. Sleep quality is measured by the question “Have you slept poorly over the past week?.” The responses of “no,”“sometimes,” and “often” are assigned scores of 0, 1, and 2, respectively. Social participation is measured by the frequency of participation in the following activities: “religious activities,” “senior university classes,” “watching TV/listening to the radio/reading books/reading newspapers/listening to opera,” “singing/playing musical instruments,” “playing mahjong/chess/cards,” and “square dancing.” Scores of 0, 1, 2, 3, and 4 are assigned for “never participated,” “a few times a year,” “at least once a month,” “at least once a week,” and “almost every day,” respectively, with a composite score range of [0, 24]. Future confidence is measured based on the statement “In my view, aging is a continuous process of loss (such as loss of health, loss of friends and relatives, loss of abilities, etc.).” The responses of “strongly disagree,” “somewhat disagree,” “neutral,” “somewhat agree,” and “strongly agree” are assigned scores of 1, 2, 3, 4, and 5, respectively.

Control variables: gender (male = 1, female = 2); age calculated as the year of the survey minus the year of birth; educational level (never attended school = 0, attended school = 1); ethnicity (Han Chinese = 1, Other ethnic groups = 2); marital Status (married = 1, unmarried = 0); religious beliefs (yes = 1, no = 0); political affiliation (Communist Party member = 1, member of a democratic party = 2, general public = 3); household size (the actual number of people living with and sharing meals with older adults respondent, including the respondent themselves); economic status (compared to those around you, how would you rate your economic situation? Better = 1, about the same = 2, worse = 3); household registration (agricultural household registration = 1, non-agricultural household registration = 2, resident household registration = 3). The specific details of variable selection and assignment are shown in Table 1.

**Table 1:** Variable selection and assignment.

Variable	Assignment	Mean	Standard deviation	Minimum	Maximum
Level of mental health	Total score on the depression scale	0.028	2.968	-6	10
Improvements to the living environment	Very satisfied = 5, somewhat satisfied = 4, neutral = 3, somewhat dissatisfied = 2, very dissatisfied = 1	3.830	0.797	1	5
Gender	Male = 1, female = 2	1.497	0.500	1	2
Age	The respondents’ chronological age	71.402	6.535	60	98
Level of education	Never attended school = 0, attended school = 1	0.773	0.419	0	1
Ethnic group	Han Chinese = 1, other ethnic groups = 2	1.058	0.234	1	2
Marital status	Married = 1, unmarried = 0	0.756	0.429	0	1
Religious beliefs	Yes = 1,no = 0	0.048	0.213	0	1
Political affiliation	Communist party member = 1, member of a democratic party = 2, general public = 3	2.920	0.392	1	3
Household size	Actual number of people living together	2.700	1.320	1	10
Economic status	Better = 1, about the same = 2, worse = 3	1.930	0.537	1	3
Household registration	Agricultural household registration = 1, non-agricultural household registration = 2, resident household registration = 3	1.528	0.590	1	3

## Empirical analysis

### Descriptive analysis

As shown in Table 1, the mean depression score for the sample of older adults was 0.028, with a standard deviation of 2.968 and a score range of [−6, 10]. This indicates that the overall depression level among Chinese older adults is relatively low and that their mental health status is generally favorable; however, the large standard deviation reflects substantial individual differences in mental health levels among older adults. The mean score for the living environment improvement variable was 3.830, with a standard deviation of 0.797, suggesting that majority of older adults in China are relatively satisfied with the environmental hygiene conditions in their communities. The small standard deviation also reveals little divergence in environmental evaluation among respondents. Regarding control variables, the mean score for the gender variable was 1.497, indicating a generally balanced gender distribution in the sample. The mean for the age variable was 71.402, with a standard deviation of 6.535 years; the age range spanned from 60 to 98 years. The sample covers the entire spectrum of older adults from younger to older age groups, demonstrating good representativeness. The mean for educational attainment was 0.773, indicating that 77.3% of respondents had received some level of education, whereas 22.7% had no schooling experience, which is consistent with the educational characteristics of China’s older adults. The mean for ethnicity was 1.058, with other ethnic groups accounting for a small proportion. Regarding marital status, 75.6% of older adults have a spouse. The mean for religious belief was merely 0.048, showing that only 4.8% of older adults hold religious beliefs. The mean for political affiliation was 2.920, demonstrating that the vast majority of older adults are ordinary citizens, while members of the Communist Party and members of democratic parties account for a small proportion. The mean household size was 2.700, with a standard deviation of 1.320 and a range of [1, 10], which reflects considerable variation in the size of older adults households. The mean economic status was 1.930, indicating that most older adults individuals considered their economic status to be comparable to that of their peers. The mean for the household registration variable was 1.528, implying that the proportions of agricultural and non-agricultural/urban household registrations were roughly equal.

### Baseline regression analysis

As shown in Table 2, Model 2 incorporated individual and household characteristics of older adults as control variables based on Model 1. The absolute values of the estimated coefficients for the independent variables decreased slightly. A possible reason for this is that the control variables partially absorbed the net effect of improvements in the living environment; however, such improvements still have a significant effect on the mental health of older adults, which was tested at the 1% significance level. This indicates that in communities with better living environments, older adults generally exhibit better mental health. Specifically, for every one-unit increase in community environment satisfaction, the level of psychological depression among older adults decreases by 0.426 points. This reveals a positive interaction between the strategic objectives of the “Healthy China” initiative and the mental health of older adults, thereby confirming Hypothesis 1.

**Table 2:** Results of the baseline regression.

Variables	Model 1	Model 2
Improvements to the living environment	−0.433^***^(0.037)	−0.426^***^(0.036)
Gender		0.169^***^(0.057)
Age		0.055^***^(0.005)
Level of educational		−0.179^**^(0.073)
Ethnic group		−0.204(0.139)
Marital status		−0.420^***^(0.069)
Religious affiliation		−0.461^***^(0.148)
Political affiliation		0.301^***^(0.068)
Household size		−0.045^**^(0.022)
Economic status		0.141^***^(0.051)
Household registration		−0.237^***^(0.051)
Constant	−0.386^*^ (0.201)	−4.454^***^(0.498)
R^2^	0.132	0.162
N	9,881	9,881
Provincial fixed effects	Yes	Yes

***, **, and * indicate significance at the 1, 5, and 10% levels, respectively; standard errors in parentheses are robust standard errors.

Females exhibit relatively poorer mental health status. The extension of life expectancy has led to an increased risk of widowhood, heightened emotional sensitivity and expressive tendency, and the long-term burden of caregiving within traditional families, all of which continue to impose psychological stress on older women (20). Meanwhile, with advancing age, the cumulative sense of loss stemming from physical deterioration, withdrawal from social roles, and higher susceptibility to chronic illnesses jointly increases the risk of depression among older adults (21). Older adults with higher education attainment, a living spouse, members of the Communist Party, religious beliefs, non-agricultural or urban household registrations, and higher economic status exhibit significantly better mental health. As a vital individual resource, a high level of education contributes to the accumulation of health capital and strengthens income-earning capacity (22), which enables people to mitigate psychological stress effectively. In the Chinese social structure, CPC (Communist Party of China) membership is often closely correlated with high social status, abundant social capital, and a strong sense of organizational belonging. Religious beliefs can help people form moral values and enhance their psychological resilience. The daily care and financial support from spouses and other family members also help enhance older adults’ resilience to resist external adverse risks and thus improving their psychological well-being. Holders of non-agricultural and resident household registrations enjoy identities advantages in accessing public services and have more stable livelihood security.

### Robustness tests

To ensure the robustness of the research findings and mitigate the impact of measurement error in the dependent variable, this paper conducted a series of robustness tests by substituting the dependent variable, replacing the estimation model, and performing placebo tests.

**Figure 2:**
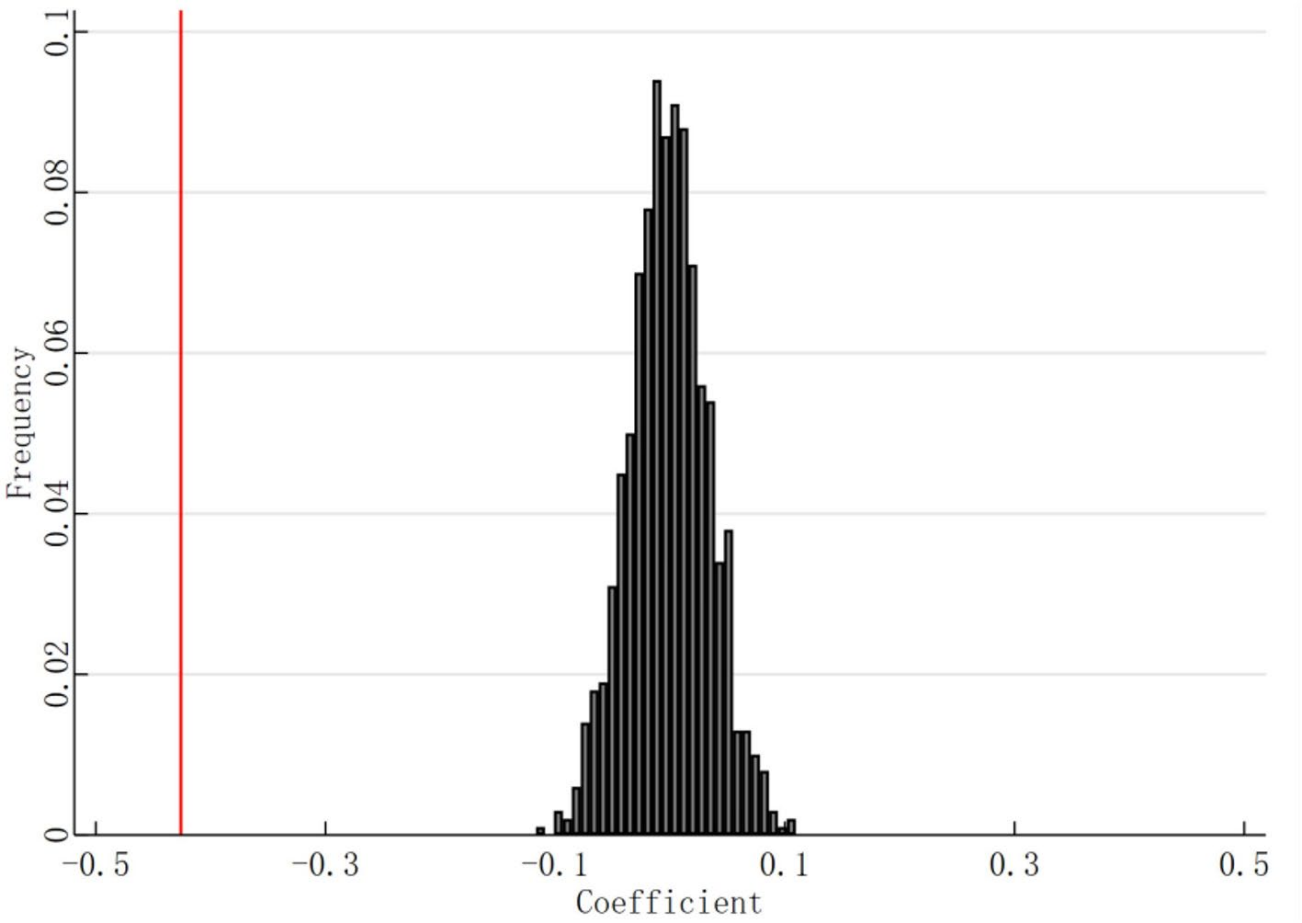
Placebo test.

1. Substitution of the dependent variable. The World Health Organization defines mental health as a state of well-being in which an individual is able to cope with life’s stresses, realize his or her own potential, and participate effectively in society (23). From a theoretical perspective, mental health is a multidimensional and complex construct that encompasses both the absence of negative psychological states such as depression and anxiety as well as the presence of positive psychological states such as life satisfaction and well-being. The CES-D scale primarily measures the frequency and intensity of depressive symptoms, corresponding to the negative dimension of mental health, whereas life satisfaction refers to an individual’s overall cognitive evaluation of life quality based on self-defined standards, representing the positive dimension of mental health (24). If consistent and significant results are obtained using life satisfaction variables that measure the same concept but represent different dimensions, this helps reduce the risk of research conclusions being subject to chance due to selection bias associated with a specific measurement tool or the limitations of a single dimension. Accordingly, this study replaces the original mental health index with life satisfaction derived from the questionnaire. Responses of very satisfied, somewhat satisfied, neutral, somewhat dissatisfied and very dissatisfied are coded as 1, 2, 3, 4 and 5, respectively. Column (1) in Table 3 shows that improvements in the living environment help reduce the risk of depression among older adults; the consistent estimation results confirm the robustness of the study’s conclusions.
2. Alternative estimation models. On the one hand, since the dependent variable is an ordered categorical variable, this study re-estimates the regression by replacing the OLS model with the ordered logit (Ologit) model. As shown in Table 3, column (2), the estimated coefficient of the core independent variable, improvements to the living environment, remains significantly negative at the 1% level. The results of the new model are largely consistent with those of the baseline regression, which further verifies the robustness of the study’s conclusions.
3. Placebo Test. To verify the robustness of the causal relationship between improvements to the living environment and mental health in older adults, this study conducts a placebo test by randomly assigning the independent variable. We randomly permute the original values of improvements to the living environment across the full sample to construct a “pseudo” -improvements to the living environment- variable, while retaining the original dependent variable and control variables unchanged. OLS regression was then performed 1,000 times. As illustrated in Figure 2, the true coefficient of improvements to the living environment is −0.426. The baseline results differ significantly from the placebo test results; the distribution of placebo coefficients obtained from 1,000 randomizations is centered around zero and approximately follow a normal distribution. This result indicates that the significant positive impact of improvements to the living environment on older adults’ mental health is unlikely to be driven by random factors or omitted variables.

**Table 3:** Robustness tests and endogeneity tests.

Variable	Replacement of the dependent variable	Alternative estimation model	Improvements to the living environment	Mental health of older adults
	(1)	(2)	(3)	
Improvements to the living environment	−0.240^***^(0.013)	−0.282^***^(0.024)	—	−1.096^***^(0.092)
Improvements to the living environment_IV	—	—	0.839^**^(0.019)	—
Control variables	Yes	Yes	Yes	Yes
Constant	−19.345^***^(3.538)	—	0.882^***^(0.144)	−1.514^**^(0.627)
First stage F	—	—	1992.92	—
Kleibergen-Paap rk LM statistic	—	—	—	884.512 [0.000]
Kleibergen-Paap rk Wald F statistic	—	—	—	1992.924 【16.38】
R^2^	0.103	0.036	—	0.134
N	11,398	9,881	9,879	9,879
Province fixed effects	Yes	Yes	Yes	Yes

***, **, and * indicate significance at the 1, 5, and 10% levels, respectively; the numbers in parentheses are robust standard errors. The values in [] correspond to the *p*-values of the LM test, and those in 【】 correspond to the 10% critical values of the Stock-Yogo test.

### Endogeneity test

The causal relationship between improvements to the living environment and the mental health of older adults may be influenced by omitted variables such as the overall community quality and institutional trust in local government. These variables are closely related to individual evaluations of environmental satisfaction and can directly or indirectly affect the level of mental health among older adults. In addition, measurement error, sample selection bias, omitted variables and bidirectional causality can easily lead to endogeneity issues. Therefore, this study adopts the average environmental satisfaction score of other respondents within the same community as an instrumental variable for the endogeneity test, which reflects the overall efforts of improvements in the living environment. Residents living in the same community share the identical public environment; thus, the environmental evaluations of neighbors are closely correlated with the respondent’s own evaluation, satisfying the correlation condition. However, others’ evaluations generally do not directly affect the respondent’s mental health, thereby also satisfying the exogeneity condition.

Column (3) of Table 3 reports the endogeneity test results estimated by the two-stage least squares (2SLS) approach. The results show that in the first stage, the F-statistic for the instrumental variable exceeded the threshold of 10, confirming a strong correlation between other respondents’ evaluations of the community environment and the respondent’s own performance evaluations, thereby effectively avoiding weak instrumental variable bias; The second-stage results suggest that improvements in the living environment still have a significant impact on the mental health of older adults, and the absolute values of the estimated coefficients of the independent variables have increased, indicating that ordinary least squares (OLS) estimates may be biased downward—that is, OLS underestimates the true impact of improvements in the living environment on the mental health of older adults. The Kleibergen-Paap rk LM statistic rejects the null hypothesis of underidentification-test at the 1% significance level, indicating that the selected instrumental variables passed the identifiability test; the Kleibergen-Paap rk Wald F-statistic exceeds the Stock-Yogo critical value at the 10% level, passing the weak instrumental variable test. The results of the instrumental variables regression are consistent with those of the baseline regression, further confirming the robustness of the conclusions.

### Mechanism analysis

The foregoing empirical results demonstrate that improvements to the living environment can effectively reduce the risk of depression among older adults, thereby enhancing their mental health. To elucidate the underlying mechanisms of these effects above and clarify the impact pathways of community environment, thereby enabling targeted policy interventions, this study introduces three mediating variables: sleep quality, social participation, and future expectations, and constructs a three-dimensional transmission mechanism framework comprising “basic physiology—behavioral interaction—time orientation.”As shown in Table 4, columns (1), (3), and (5) present the regression results of improvements to the living environment on older adults’ sleep quality, social participation, and future expectations, respectively. The coefficients of improvements to the living environment are −0.047, 0.309 and −0.098, all of which pass the significance test at the 1% level. This indicates that improvements to the living environment can significantly improve older adults’ sleep quality, increase their social participation, and maintain their confidence for the future. Columns (2), (4), and (6) reveal that after incorporating three mediating variables into the main regression, their coefficients were 1.461, −0.076, and 0.090, respectively, all passing the significance test at the 1% level. This indicates that sleep quality, social participation, and future confidence can reduce the risk of depression among older adults and improve their psychological well-being. It can thus be concluded that sleep quality, social participation and confidence in the future do exert significant mediating effects between improvements in the living environment and mental health in older adults; Hypothesis 2 has been supported.

**Table 4:** Mediation effect test results.

Variable	(1)	(2)	(3)	(4)	(5)	(6)
	Sleep quality	Mental health in older adults	Social engagement	Mental health in older adults	Confidence in the future	Mental health in older adults
Improvements to the living environment	−0.047^***^ (0.009)	−0.359^***^ (0.035)	0.309^***^ (0.042)	−0.402^***^ (0.036)	−0.098^***^ (0.029)	−0.489^***^ (0.044)
Sleep quality		1.461^***^ (0.041)				
Social participation				−0.076^***^ (0.009)		
Future confidence						0.090^***^ (0.018)
Control variables	Yes	Yes	Yes	Yes	Yes	Yes
Constant	0.581^***^ (0.055)	−0.705^**^ (0.279)	3.947^***^ (0.264)	−3.771^***^ (0.503)	4.097^***^ (0.247)	−0.795^**^ (0.313)
R^2^	0.067	0.244	0.201	0.168	0.078	0.133
N	9,881	9,881	9,881	9,881	7,394	7,394

***, **, and * indicate significance at the 1, 5, and 10% levels, respectively; values in parentheses are robust standard errors.

To further verify the reliability of the mechanism results, this study performs Bootstrap tests (500 random draws) using Stata statistical software to examine the mediating effects of sleep quality, social participation and future confidence on the impact of improvements in the living environment on the mental health of older adults. As reported in Table 5, the mediation coefficients of the sleep quality, social participation, and confidence in the future are −0.062, −0.041, and −0.005, respectively. None of the 95% confidence intervals include zero, and the indirect effects passed the significance test, demonstrating the robustness of the proposed mechanism.

**Table 5:** Bootstrap test results.

Variable	Observed coefficient	Bootstrap std. err	*Z*	*P*	95% Conf. interval
Sleep quality	−0.062^***^	0.013	−4.84	0.000	[−0.0864022,-0.0365986]
Social participation	−0.041^***^	0.006	−6.90	0.000	[−0.0522320,-0.0291351]
Future confidence	−0.005^**^	0.043	−2.17	0.030	[−0.0100312,-0.0005170]

** and *** indicate significance at the 5 and 1% levels, respectively.

### Heterogeneity analysis

#### Heterogeneity by urban–rural location

There are considerable disparities in the living environments between urban and rural areas; therefore, the extent and direction of the impact of improvements in the living environment on the mental health of older adults may differ across regins. To further examine the urban–rural regional differences in such effects, this study conducted grouped regressions for urban and rural older adults subsamples separately.

Columns (1) and (2) in Table 6 show that the estimated coefficients for improvements in the living environment are −0.348 and −0.534 for urban and rural older adults, respectively, both pass the significance test at the 1% level. In other words, the higher level of environmental satisfaction, the lower depressive scores among older adults, indicating that improvements to the living environment have a positive effect on their mental health. However, the absolute values of the estimated coefficients for rural older adults were noticeably higher than those for urban older adults, suggesting that improvements in the living environment have a stronger positive impact on the mental health of rural older adults than on that of urban older adults. The impact of improvements in the living environment on older adults’ mental health varies across urban and rural areas, with rural older adults benefiting more from such improvements.

**Table 6:** Heterogeneity test.

Variable	Urban (1)	Rural (2)	Self-reported health: good (3)	Self-reported health: poor (4)	High income (5)	Low income (6)
Improvements to the living environment	−0.348^***^ (0.049)	−0.534^***^ (0.055)	−0.524^***^ (0.054)	−0.188^***^ (0.047)	−0.514^***^ (0.046)	−0.274^***^ (0.060)
Control variables	Yes	Yes	Yes	Yes	Yes	Yes
Constant	−6.514^***^ (0.670)	−2.707^***^ (0.969)	−1.922^***^ (0.735)	−3.728^***^ (0.677)	−0.981 (0.721)	−8.177^***^ (0.815)
R^2^	0.193	0.159	0.207	0.149	0.156	0.161
N	5,587	4,294	4,847	5,024	5,892	3,989

***indicate significance at the 1% levels; standard errors are shown in parentheses.

Rural areas rely more heavily on natural resources, and rural residents maintain frequent direct interactions with farmland, forests and other natural environmental elements (25). Accordingly, improvement to the living environment exerts a more immediate psychological impact on rural older adults. In addition, rural older adults generally have lower income levels, inadequate social security coverage and relatively scarce medical resources, and their social networks are centered on kinship and geographical relation. Consequently, environmental improvements are more likely perceived as a form of community public welfare, which strengthens community cohesion and a sense of collective efficacy. Through the social support mechanism, it improves older adults’ mental health and directly raises their life satisfaction and psychological security. By contrast, urban older adults individuals have relatively higher income levels, diverse recreational options, and more sophisticated public infrastructure and mental health services, which effectively buffer the negative impact of environmental issues on mental health, thereby diminishing the marginal effects of environmental improvements.

#### Heterogeneity in self-reported health

To examine whether the impact of improvements to the living environment on mental health varies with older adults’ baseline health status, this study divides the sample into two groups based on baseline health status: those with relatively good self-reported health and those with relatively poor self-reported health. Health status was measured using the questionnaire item “How would you evaluate your current physical health?” Responses of “very healthy” and “fairly healthy” were combined into the category “healthy” and assigned a value of 1, while all other responses were assigned a value of 2. As shown in columns (3) and (4) of Table 6, the estimated coefficient of improvements to the living environment is −0.524 in the “good self-reported health” group and −0.188 in the “poor self-reported health” group, with both being significant at the 1% level. This indicates that regardless of older adults’ self-reported health, improvements to the living environment can enhance their mental health, though the beneficial effect is notably stronger among the healthier group.

Self-reported health, as a critical indicator of an individual’s overall physiological function and vitality, reflects that healthy older adults possess both superior physical function and optimistic psychological capital. As community environments become increasingly clean, well-landscaped, and enriched with public activity spaces through governance, healthy older adults are more capable and willing to step out of their homes to participate in outdoor activities. They can therefore directly derive positive experiences such as physical and mental pleasure, stress relief, and social fulfillment from these improvements, and more inclined to view a good living environment as an additive resource that can be actively utilized to enhance their quality of life. Good physical health and high-quality living environments are two mutually reinforcing and synergistic advantageous resources. Healthy older adults can fully exploit environmental capital, which in turn improves their mental well-being, forming a Matthew effect of cumulative advantage and generating positive psychological feedback to residential environment improvement. By contrast, frail older adults are restricted by chronic pain or several diseases. While enduring health-related pressure, they suffer from higher levels of anxiety, helplessness and depressive emotions. Their psychological resources are often devoted to coping with and managing their own health issues, so improvements to their environment cannot effectively substitute or compensate for impaired health functions. Consequently, the psychological benefits of improving the living environment are substantial diminished, resulting in an inequity whereby environmental governance disproportionately benefit those who are already in good health.

#### Income heterogeneity

Income is a core indicator of older adults’ socioeconomic status and profoundly shapes individual capabilities to cope with environmental challenges and access health well-being. Accordingly, this study divides the older adults sample into high-income and low-income groups based on the average total personal income over the past year. The results of the income heterogeneity test reveal a concerning trend: the beneficial effect of residential environment improvement on mental health differs substantially between high-income and low-income older adults. As shown in columns (5) and (6) of Table 6, the estimated coefficient for improvements to the living environment is −0.514 for high-income older adults, and it passes the significance test at the 1% level. A one-unit increase in environmental satisfaction reduces depressive scores by 0.514 points among high-income older adults, with a much larger improvement in psychological well-being compared with their low-income counterparts. The impact of improvements in the living environment on older adults’ mental health varies depending on income level.

On the one hand, high-income older adults can convert superior objective living environments into tangible psychological well-being by virtue of their economic capital, deeply immersing themselves in and enjoying the benefits of improvements to the living environment. Conversely, even when living in the same community environment, low-income older adults are constrained by financial difficulties. They cannot afford additional expenditure on leisure activities and are confined to free basic public spaces, which diminishes the marginal benefits of environmental improvement. On the other hand, according to Maslow’s hierarchy of needs, once the basic survival needs of high-income older adults are fully satisfied, they tend to pursue higher-level quality of life and self-worth, and thus have higher expectations and a keener perception of environmental quality. The improvements in living environment precisely meets high-income older adults’ pursuit of a high-quality life, and brings them immense psychological satisfaction. In contrast, low-income old adults reoccupied with sustaining daily livelihoods, with their primary concerns centered on basic needs including food security, medical care and housing. Consequently, their expectations regarding environmental quality are relatively low. Faced with heavy life pressures, the psychological comfort derived from environmental improvements is relatively limited.

## Conclusion and discussion

Based on the 2020 China Longitudinal Aging Social Survey, this paper comprehensively investigates the actual impact of improvements in the living environment on the mental health of older adults. Descriptive analysis reveals that, currently, the overall level of despressive symptoms among older adults in China are generally low, their mental health status is generally favorable, and they are relatively satisfied with the environmental hygiene conditions in their communities. After controlling for individual characteristics and family factors among older adults, empirical analysis demonstrates that the benefits of improvements in the living environment do indeed extend beyond the realm of “physical health” and have a positive effect on the mental health of older adults. For every one-unit increase in community environmental satisfaction reduces older adults’ depression scores by 0.426 points. To ensure the reliability of the study’s conclusions, robustness tests were conducted using methods such as replacement variables, model switching, placebo tests, and instrumental variables. The test results were consistent across all methods. In particular, the results from the instrumental variables approach even suggest that ordinary least squares (OLS) tended to underestimate the true effect of improvements in the living environment. On the other hand, the study shed light on the “environment–psychological” linkage, revealing that three parallel pathways—“basic physiology,” “behavioral interaction,” and “time orientation”—collectively constitute a multidimensional transmission mechanism through which improvements in the living environment influence the mental health of older adults. However, the psychological benefits of these improvements are unevenly distributed, with their positive effects being more pronounced among three specific groups of older adults: those in rural areas, those with high-income, and those who self-reported good health.

This paper extends the research scope of improvements to the living environment from traditional ecological and physiological health dimensions to the domain of mental health. It is of great significance for leveraging the positive effects of such improvements on mental health and promoting the in-depth integration between the “Beautiful China” and “Healthy China” strategies. First, improvements to the living environment helps reduce environmental stressors. By eliminating negative stimuli such as pollution, noise and safety hazards, it can effectively alleviate the chronic stress responses caused by older adults long-term exposure to adverse environments and reduce sleep problems resulting from difficulty falling asleep and sleep interruptions. This interrupts the physiological link between adverse environments and the induction of negative emotions, thereby reducing the risk of psychological issues such as depression and anxiety among older adults at a fundamental level. Second, improvements to the living environment helps optimize public spaces and provides the essential venues for older adults to engage in neighborhood interactions, cultural and recreational activities, and other pursuits. This effectively reduces the likelihood of social isolation among older adults and encourages their active participation in society, thereby helping them build and activate social support networks. As a result, older adults can access greater external support when coping with negative life events, continuously enhancing their psychological resilience. Finally, a safe, stable, livable and age-friendly living environment significantly enhances older adults’ sense of security and fulfillment in their daily lives. This, in turn, fosters positive expectations for their later years, enriches their positive cognitive and emotional resources, and builds a protective zones for mental health at the cognitive level, thereby fundamentally reducing the occurrence of negative psychological issues. Overall, the protective effect of perceived residential environment on older adults’ mental health identified in this study is consistent with the findings from comparable international studies. Empirical studies on community-based older adults health in the United States and Europea demonstrates that clean and well-organized community environments help reduce the risk of depression among older adults (26, 27). These studies confirm that there is a general theoretical foundation underlying the link between living environment and older adults mental health; the findings of this study, in turn, supplement the global consensus in this field with empirical evidence from a large developing country. On the other hand, the findings regarding urban–rural heterogeneity in this paper differ from existing research in Western countries. Developed countries generally have a higher degree of equity in public services between urban and rural areas, with smaller disparities in environmental sanitation infrastructure. In contrast, China’s long-standing urban–rural dual structure has led to inadequate environmental governance in rural communities compared with urban areas (28); consequently, the marginal health gains from improving the rural living environment are significantly greater than those for urban population. This finding adds to the empirical evidence regarding urban–rural disparities in environmental health effects, indicating that health benefits derived from the living environment vary hugely depending on the institutional context; consequently, policy formulation for China cannot simply replicate the conclusions of studies from other countries. So, how can we take full advantage of the psychological benefits of improving the living environment? On the one hand, it is necessary to enrich the evaluation system for living environment improvements. Within top-level policy designs such as the 15th Five-Year Plan for Ecological and Environmental Protection and the “Healthy China” Implementation Plan, the mental health benefits for residents should be incorporated into the policy evaluation framework for living environment improvements, thereby driving the in-depth development of coordinated “environment–health” governance. Relying on large-scale cohort studies such as the nationwide longitudinal social survey of older adults, a dynamic linked database combining environmental monitoring data and residents’ mental health indicators should be established to quantitatively measure the contribution of major environmental projects to older adults psychological well-being. Adhering to a people-centered development philosophy, this study recommends introducing analyses of psychological benefits and costs, monetizing hidden social benefits—such as the avoided burden of mental illness and savings in mental health service expenditures—to comprehensively evaluate the overall returns of investments in improving the living environment. This will help departments including environmental protection, health, housing and civil affairs form a coordinated policy effort to promote the all-round development of individuals. In addition, the government should precisely identify and prioritize support for vulnerable groups who stand to gain the most from improvements in the living environment in terms of psychological well-being, and ensure that policy resources are prioritized for disadvantaged older adults to guarantee environmental heath equality. In rural areas, the government should align with the Rural Revitalization Strategy, promoting older adults mental health should be set as a core objective of the rural living environment improvement initiative. Local government should focus investment on projects—such as village greening, public activity squares, and mutual-aid facilities for older adults—that both improve the environment and directly foster social interaction. Urban communities should prioritize barrier-free design and inclusive planning to lower the threshold for accessing environmental welfare. Furthermore they should explore integrated models that combine improvements to the living environment with community support to help vulnerable older adults overcome physical limitations or financial constraints. By providing public spaces that support various forms of social interaction, the governments can build age-friendly communities, ensure that the health benefits of improvements to the living environment are equitably shared by all older adults, and bridge the mental health gaps among this demographic.

This study also has the following limitations: First, at the research design level, there are limitations in causal inference; cross-sectional data can only capture the association between community environmental hygiene satisfaction and levels of depression among older adults at a single time point and cannot support dynamic longitudinal tracking studies over time. Furthermore, although this study has progressively controlled for various variables—such as sociodemographic characteristics and household economic status—there may still be unobserved individual traits that simultaneously influence both environmental perceptions and mental health status. Therefore, the reliability of our findings needs to be further verified by future longitudinal panel data and quasi-experimental designs. Second, additional latent mediating factors may exist in the association between residential environment improvement and older adults mental health, meaning the underlying mechanisms remain to be further explored and expanded in subsequent research.

## Data Availability

The raw data supporting the conclusions of this article will be made available by the authors, without undue reservation.
